# Antibacterial and anti-inflammatory activities of an extract, fractions, and compounds isolated from *Gochnatia pulchra* aerial parts

**DOI:** 10.1590/1414-431X20154410

**Published:** 2015-07-21

**Authors:** R. Lucarini, M.G. Tozatti, M.L.A. Silva, V.M.M. Gimenez, P.M. Pauletti, M. Groppo, I.C.C. Turatti, W.R. Cunha, C.H.G. Martins

**Affiliations:** 1 Núcleo de Pesquisas em Ciências Exatas e Tecnológicas, Universidade de Franca, Franca, SP, Brasil; 2 Centro Universitário Claretiano, Batatais, SP, Brasil; 3 Departamento de Biologia, Faculdade de Filosofia, Ciências e Letras de Ribeirão Preto, Universidade de São Paulo, Ribeirão Preto, SP, Brasil; 4 Departamento de Física e Química, Faculdade de Ciências Farmacêuticas de Ribeirão Preto, Universidade de São Paulo, Ribeirão Preto, SP, Brasil

**Keywords:** *Gochnatia pulchra*, Anti-inflammatory activity, Antibacterial activity, Medicinal plants, Proinflammatory cytokines

## Abstract

This paper reports on the *in vitro* antibacterial and *in vivo* anti-inflammatory properties of a hydroethanolic extract of the aerial parts of *Gochnatia pulchra* (HEGP). It also describes the antibacterial activity of HEGP fractions and of the isolated compounds genkwanin, scutellarin, apigenin, and 3,5-O-dicaffeoylquinic acid, as evaluated by a broth microdilution method. While HEGP and its fractions did not provide promising results, the isolated compounds exhibited pronounced antibacterial activity. The most sensitive microorganism was *Streptococcus pyogenes*, with minimum inhibitory concentration (MIC) values of 100, 50 and 25 µg/mL for genkwanin and the flavonoids apigenin and scutellarin, respectively. Genkwanin produced an MIC value of 25 µg/mL against *Enterococcus faecalis*. A paw edema model in rats and a pleurisy inflammation model in mice aided investigation of the anti-inflammatory effects of HEGP. This study also evaluated the ability of HEGP to modulate carrageenan-induced interleukin-1 beta (IL-1β), tumor necrosis factor alpha (TNF-α), and monocyte chemoattractant protein-1 (MCP-1) production. Orally administered HEGP (250 and 500 mg/kg) inhibited carrageenan-induced paw edema. Regarding carrageenan-induced pleurisy, HEGP at 50, 100, and 250 mg/kg diminished leukocyte migration by 71.43%, 69.24%, and 73.34% (P<0.05), respectively. HEGP suppressed IL-1β and MCP-1 production by 55% and 50% at 50 mg/kg (P<0.05) and 60% and 25% at 100 mg/kg (P<0.05), respectively. HEGP abated TNF-*α* production by macrophages by 6.6%, 33.3%, and 53.3% at 100, 250, and 500 mg/kg (P<0.05), respectively. HEGP probably exerts anti-inflammatory effects by inhibiting production of the pro-inflammatory cytokines TNF-α, IL-1β, and MCP-1.

## Introduction

Trees belonging to the genus *Gochnatia* (Asteraceae) have found wide application in folk medicine (1,2). The 70 species that constitute this genus mainly originate in North and South America, from Mexico to Argentina (3). Few studies on the chemical constituents of *Gochnatia* exist. To date, scientists have investigated only 14 species and identified sesquiterpene lactones, sesquiterpenes, diterpenes, triterpenoids, flavonoids, and coumarins as their major compounds (4). The Brazilian population has used the aerial parts of *Gochnatia pulchra*, known as “cambara”, to treat infections, coughs, bronchitis, inflammation, stomach pain, headaches, general pain, and rheumatism, and this species also serves as a general tonic. Despite the lack of chemical and biological studies on *G. pulchra*, researchers have detected sesquiterpene lactones, dimeric guaianolides, bisabolenes, diterpenes, triterpenes, and coumarins in *Gochnatia polymorpha* (subspecies polymorpha or unspecified) ([Bibr B05] [Bibr B06] [Bibr B07] 58). Although active extracts of *G. polymorpha* leaves contain flavonoids and phenolic compounds, these constituents do not account for the antibacterial and anti-inflammatory activities of this plant (9).

The present study aimed to evaluate the scientific basis for the traditional use of *G. pulchra* by assessing its antibacterial and anti-inflammatory properties.

## Material and Methods

### Plant material

Aerial parts of *G. pulchra* (Spreng.) Cabrera (Asteraceae family) were collected in March 2008 in Reserva de Jataí, near the city of Luis Antônio, state of São Paulo, Brazil. The plant material was identified by Dr. Milton Groppo. A voucher specimen (SPFR 137001) was deposited in the Herbarium of Departamento de Biologia, Faculdade de Filosofia, Ciências e Letras da Universidade de São Paulo (Herbarium SPFR).

### Plant extract preparation and partitioning

The aerial parts of *G. pulchra* were dried in a stove with circulating air (40°C) and powdered in a blender. The powder (900 g) was exhaustively extracted with ethanol/H_2_O (8:2 v/v) by maceration at room temperature (25°C), followed by filtration. The filtered extract was concentrated under reduced pressure, affording an aerial extract (HEGP; 45.8 g). A part of HEGP (30.0 g) was suspended in ethanol/H_2_O (4:1 v/v) and submitted to sequential partition, to produce *n*-hexane (HF), dichloromethane (DF), ethyl acetate (EF), and aqueous (AQF) fractions in yields of 9.0, 12.4, 2.0, and 1.5 g, respectively. All of the fractions were transferred to pre-weighed vials and kept in a refrigerator for later use in biological assays.

### Compound isolation

The DF fraction (10.0 g) was chromatographed through a silica gel column, using increasing proportions of hexane, ethyl acetate, ethanol, and mixtures thereof as eluents, to give 15 fractions. Fraction 7 (F-7) contained a yellow amorphous solid, identified as the flavone genkwanin (1). Fraction 10 (F-10) was analyzed by RP-HPLC with the aid of a Shim-pack phenyl column (250×20 mm; 5 µm; pore diameter: 100 Å; Shimadzu, Japan), using solutions of H_2_O containing 0.1% AcOH (A) and MeOH (B) as eluents (isocratic method: 50% B for 40 min; flow rate: 9 mL/min), which yielded two other flavones, scullaterin (2) and apigenin (3).

The EF fraction (1.0 g) was chromatographed on a column containing Sephadex LH-20, using methanol as the eluent, and 6 fractions were obtained. Fraction 5 was analyzed by RP-HPLC on a Shim-pack ODS column (Shimadzu; 250×20 mm; 5 µm; pore diameter: 100 Å), using solutions of H_2_O containing 0.1% AcOH (A) and MeOH (B) as eluents (isocratic method: 45% B for 30 min; flow rate: 9 mL/min). This procedure led to isolation of the compound 3,5-O-dicaffeoylquinic acid (4).

### Gas chromatograph-mass spectrometry (GC-MS) analysis

HF was analyzed on a QP-2010 GC-MS system (Shimadzu) equipped with a split injector operating at 250°C. DB-5MS (5% phenyl and 95% dimethyl arylene siloxane; 30 m×0.25 mm×0.25 µm; linear rate: 39 cm/s) and DB-17MS (50% phenyl and 50% dimethyl arylene siloxane; 30 m×0.25 mm×0.25 µm; linear rate: 44.4 cm/s) capillary columns were employed. In the case of DB-5MS, the oven temperature was programmed to increase from 100°C to 290°C within 30 min, and helium was used as the carrier gas at an average column flow rate of 1.10 mL/min. For DB-17MS, the oven temperature was programmed to increase from 120°C to 260°C within 5 min, from 260°C to 280°C within 9 min, and from 280°C to 290°C within 25 min. Helium was also used as the carrier gas, with an average column flow rate of 1.4 mL/min. Triterpenes were identified by comparison of the relative retention (RR) values of the samples with the RR values of the standard triterpenes and by comparison of their mass spectra with literature data (10,11). Authentic standards available in our laboratory were also co-eluted with HF to confirm the identity of the components.

### Standard triterpenes

The certified standard triterpenes employed in the GC-MS analysis were purchased from Sigma-Aldrich (USA).

### Structural identification

The chemical structures of the compounds were determined by spectroscopic methods. ^1^H-NMR (400 MHz) and ^13^C-NMR (100 MHz) spectra were recorded on a Bruker DPX-400 spectrometer (Bruker Corporation, USA) in dimethylsulfoxide (DMSO)-d_6_ or CDCl_3_, with tetramethylsilane as the internal standard. High-resolution ESI-MS was recorded on a Micromass Q-Tof (quadrupole time-of-flight) mass spectrometer (Bruker Corporation).

### Antibacterial activity

An adapted microtiter dilution assay (12) was used to determine the minimum inhibitory concentration (MIC) of the phytochemical compounds. Standard reference bacterial strains (*Staphylococcus aureus*: 25923; *Streptococcus pyogenes*: 19615; *Enterococcus faecalis*: 19433; *Escherichia coli*: 14948; *Salmonella* Choleraesuis: 10708; and *Pseudomonas aeruginosa*: 27853) from the American Type Culture Collection (ATCC, USA) were employed. Samples were dissolved in DMSO (Synth, Brazil) at 1 mg/mL, followed by dilution in Mueller Hinton broth (Difco, USA). Concentrations ranging from 400.0 to 1.0 µg/mL were achieved in microplates (96 wells). The final DMSO content was 5% (v/v), and a solution of DMSO at this concentration was used as the negative control. The inoculum was adjusted for each organism to yield a cell concentration of 5×10^5^ colony-forming units (CFU)/mL, according to the guidelines of the Clinical and Laboratory Standards Institute. One inoculated well was included as a control for whether the broth was adequate for organism growth, and one non-inoculated well free of antimicrobial agents was employed to ensure medium sterility. All MIC values were determined in triplicate. After incubation for 24 h in an incubator at 37°C, the MIC was evaluated as the lowest concentration of the tested substance that inhibited growth of the bacterial strain. Penicillin and streptomycin were used as standard antibiotics (positive controls).

### Animals

Adult (60 to 70 days) male Wistar rats weighing 180-220 g (n=178) and adult male Swiss mice weighing 25-35 g (n=42), obtained from the Central Animal Housing Facility of the University of Franca, were housed under controlled light (12-h/12-h light/dark cycle; lights on at 6:00 am) and temperature (23±1°C) conditions with access to water and food *ad libitum*. The animals were allowed to acclimatize to the housing facilities for at least 1 week before the experiments. All procedures used complied with the guidelines advocated by the Ethical Treatment of Animals in Applied Animal Behavioral Research and with the principles of the Brazilian College of Animal Experimentation. The Ethics Committee of the University of Franca approved the study protocol (#023/08A). To test the anti-inflammatory activity of the target compounds, doses of 50, 100, 250, and 500 mg/kg suspended in vehicle (1% Tween-20 suspension in distilled water) were administered to the animals. Dexamethasone and indomethacin in vehicle were used as reference drugs and were orally administered in a volume equivalent to 5 and 10 mL/kg body weight of the animals, respectively.

### Anti-inflammatory activity

Paw edema was induced according to Winter et al. (13). Rats were randomly divided into five groups (n=6 per group), and orally pretreated with vehicle (0.9% saline plus 1% Tween-80) at 0.1 mL/100 g in the negative control group, HEGP (100, 250, and 500 mg/kg), or the reference anti-inflammatory agent indomethacin (5 mg/kg). After 60 min, edema was induced by injection of 0.1 mL of carrageenan (100 µg/paw) in saline into the right hind paw. The left hind paw was used as a control, and received injection of vehicle (saline; 100 µL). Inflammation was quantified by measuring the volume (mL) displaced by the paw at 0, 1, 3, and 4 h after carrageenan injection, using a plethysmometer (Model 7140; Ugo Basile, Italy). Results are reported as the difference in the volumes (mL) of the right and left paws at each time point (14).

### Leukocyte and neutrophil migration into the peritoneal cavity in mice

Leukocyte and neutrophil migration into the peritoneal cavity was investigated as previously described (15). Mice (n=6 per group) were orally pretreated with HEGP (50, 100, 250, and 500 mg/kg) or vehicle (0.1 mL/10 g) at 60 min before intraperitoneal injection of carrageenan (500 mg/cavity, 0.5 mL) or sterile saline (0.5 mL) into the peritoneal cavity. Dexamethasone (10 mg/kg, oral administration) was used as the reference anti-inflammatory drug. At 3 h after carrageenan injection, the animals were euthanized by cervical displacement. Immediately afterward, a volume of 3 mL of phosphate-buffered saline (PBS) containing ethylenediamine tetraacetic acid (1 mM) was injected into the peritoneal cavity, and the numbers of total cells (leukocytes) and differentiated cells (neutrophils) were counted. To perform the total count, peritoneal fluid (20 µL) was diluted in Turk solution (0.4 mL). Counting was accomplished with a Neubauer cell counting chamber (30×70 mm and 4 mm thickness; Celeromics, USA), and the results are reported as the number of leukocytes per milliliter of peritoneal washing fluid. Next, part of the peritoneal fluid was centrifuged at 78.4 *g* for 10 min. The supernatant was re-suspended, and the neutrophils were counted. The cells were stained with hematoxylin-eosin and counted under a light microscope (Eclipse, Nikon, USA), using an oil immersion objective. The number of differentiated cells was calculated as the percentage of differentiated cells found in the total number of cells (total of 100 cells).

### Cytokine quantification

IL-1β, TNF-α, and MCP-1 levels in local tissue homogenate supernatants were measured using enzyme-linked immunosorbent assay (ELISA) kits - Rat IL1β, Rat MCP-1 (CCL2), Rat TNF-α (BioSource International Inc., USA) - in accordance with the procedures recommended by the manufacturer. In brief, IL-1β levels were measured by pipetting 50 µL of sample and 50 µL of standard dilution buffer into the wells of a microtiter plate coated with an antibody specific for rat IL-1β, followed by incubation for 3 h at 37°C. After two 10-min washes with PBS, a biotinylated anti-rat IL-1β antibody was added and incubated at ambient temperature for 1 h. Streptavidin-peroxidase HRP was then added and incubated for 30 min, to allow binding of the enzyme to the biotinylated antibody. After unbound enzyme was removed by two more 10-min washes with PBS, color was developed by adding the stabilized chromogen tetramethyl benzidine, followed by a stop solution. Finally, the absorbances were measured with an automated Coulter microplate reader (T890, Coulter Electronics, USA) at 450 nm, and the IL-1β protein levels were quantified by comparing the samples with a standard curve generated from the kit. The results are reported as IL-1β concentrations (pg/mg protein). The same procedure was used to assay TNF-α and MCP-1 with the aid of antibodies specific for these cytokines. The assays were performed by an investigator blinded to the assignment of the treatment groups.

### Statistical analysis

Data were analyzed using GraphPad Version 4.0 (GraphPad Software Inc., USA), and are reported as means±SE. The statistical significance of differences between groups was evaluated by analysis of variance (ANOVA).

## Results

### Phytochemical analysis

**Figure 1:**
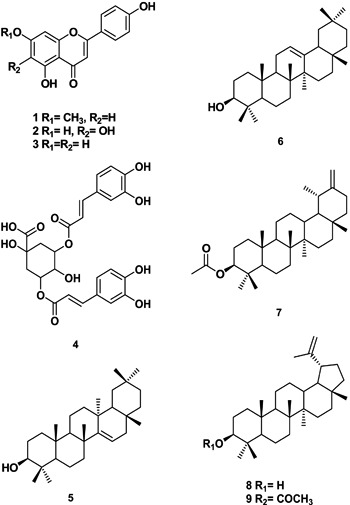
Chemical structures of the compounds identified in *Gochnatia pulchra*: genkwanin (1); scutellarin (2); apigenin (3); 3,5-O-dicaffeoylquinic acid (4); epitaraxerol (5); β-amyrin (6); taraxasterol acetate (7); lupeol (8); and lupeol acetate (9).

Phytochemical investigation of the DF fraction revealed the presence of three flavonoids, genkwanin, scutellarin, and apigenin (Figure 1), as the major constituents. This is the first report of these compounds in *G. pulchra*. Phytochemical investigation of the EF fraction led to the isolation of 3,5-O-dicaffeoylquinic acid. ^1^H-NMR and ^13^C-NMR data analyses and comparisons with literature data helped to establish the chemical structures of the isolated compounds as genkwanin (16), scutellarin (17), apigenin (18), and 3,5-O-dicaffeoylquinic acid (19). GC-MS analysis of the HF fraction aided the identification of five compounds, namely epitaraxerol, β-amyrin, taraxasterol acetate, lupeol, and lupeol acetate (Figure 1), as evidenced by comparisons of their relative retention (RR) values with those of authentic standards obtained with the DB-5MS and DB-17MS columns (Table 1) and by comparison of their mass spectra with literature data (10,11). Co-elution of authentic standards available in our laboratory with HF helped to confirm the identity of these components.

**Table:**
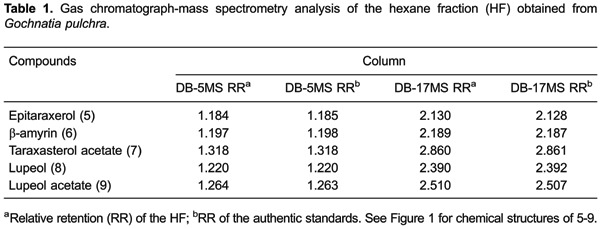

### Antibacterial activity

HEGP and the HF, DF, EF, and AQF fractions displayed low activity against the *E. faecalis*, *E. coli*, *S*. Choleraesuis, and *P. aeruginosa* strains examined (Table 2). HF, DF, and AQF were slightly more active against *S. aureus* (200 µg/mL) and *S. pyogenes* (200 µg/mL). Genkwanin inhibited the growth of *S. pyogenes* and *E. faecalis*, with MIC values of 100 and 25 µg/mL, respectively (Table 2), followed by the flavonoids scutellarin and apigenin, with an MIC value of 25 and 50 µg/mL against *S. pyogenes*, respectively. The similar MIC values obtained for the compounds may have resulted from their similar chemical structures. Under the concentrations and conditions tested, none of the compounds were active against Gram-negative bacterial strains.

**Table:**
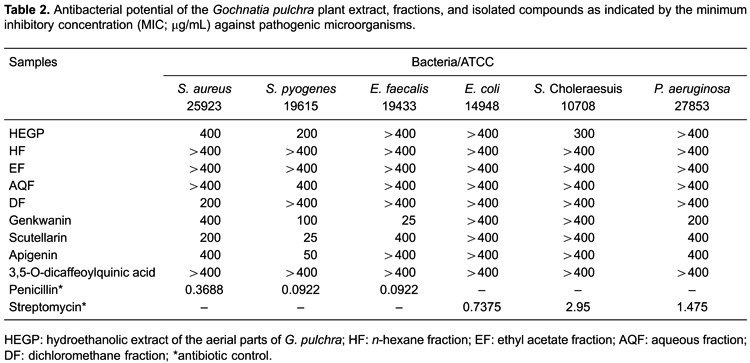

### Anti-inflammatory activity

**Figure 2:**
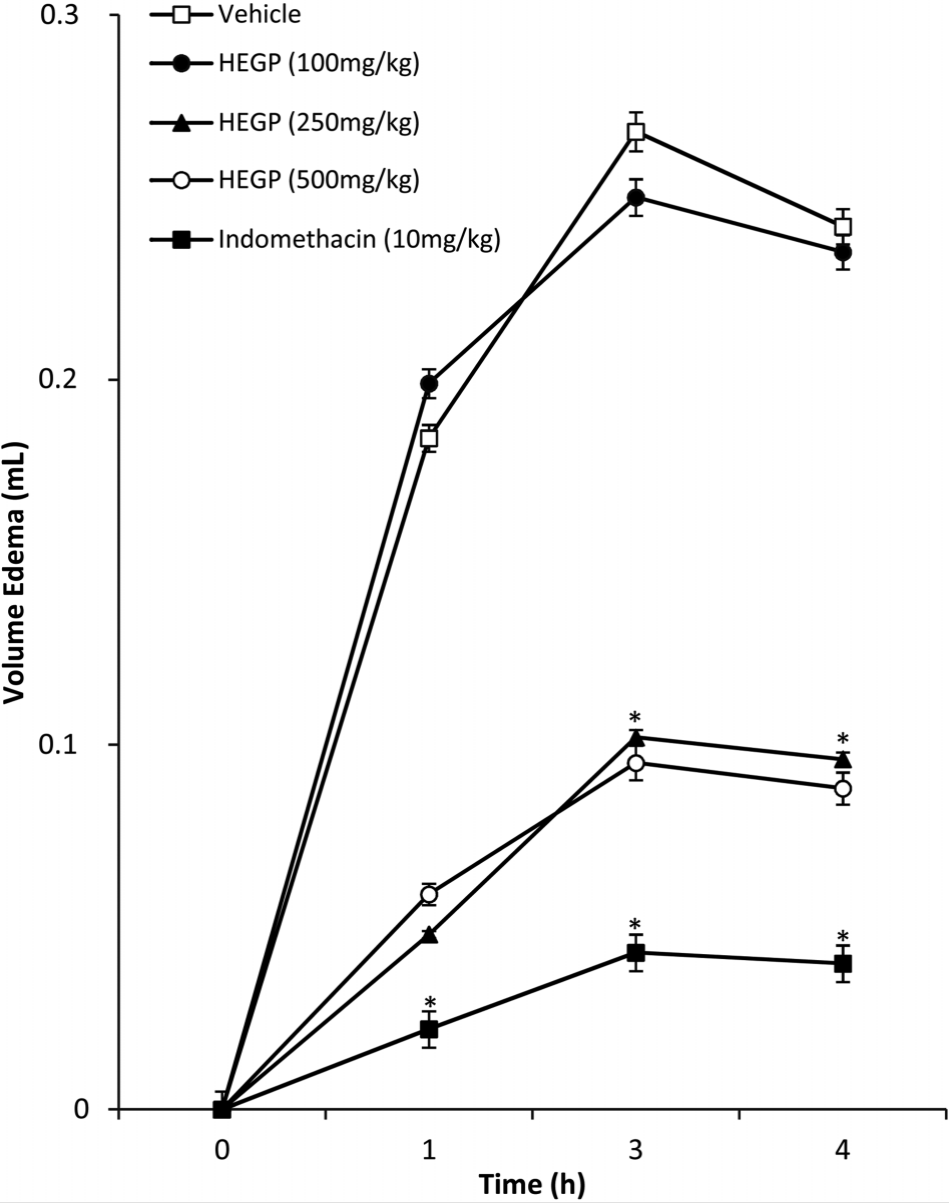
Effects of oral administration of the hydroethanolic extract of *Gochnatia pulchra* (HEGP; 100, 250, and 500 mg/kg) or indomethacin (5 mg/kg) on rat paw edema induced by intraplantar carrageenan injection (0.1 mg/paw). Data are reported as means±SE of 6 animals. *P<0.05, compared to the vehicle control (one-way ANOVA).

Figure 2 presents the results for the effects of orally administered HEGP in the carrageenan-induced rat paw inflammation assay. Compared with the negative control (vehicle), HEGP at doses of 100, 250, and 500 mg/kg inhibited edema by 9.32%, 61.95%, and 64.56%, respectively, while the standard drug indomethacin reduced paw edema by 74.85%. The comparison of the effects was performed at 3 h, when the edema peaked. The anti-inflammatory activity of the extracts was dependent on the HEGP concentration (250 and 500 mg/kg), and the activity improved as the concentration increased. HEGP displayed significant anti-inflammatory effects compared with the negative control (P<0.05, one-way ANOVA).

### Leukocyte and neutrophil migration into the peritoneal cavity in mice

Regarding the effects on pleurisy, the tested doses significantly reduced the volume of the exudate and the numbers of total leukocytes and neutrophils (Table 3). Inhibition of leukocyte migration only occurred at a dose of 50 mg/kg (44.4% inhibition). Dexamethasone (positive control) reduced the volume of the exudate leukocyte migration by 55.5%. Compared with the control, the numbers of polymorphonuclear and mononuclear cells did not differ significantly. Statistical analyses showed that the anti-inflammatory effect of HEGP was only significant at 50 mg/kg, while the other evaluated concentrations showed no significant activity.

**Table:**
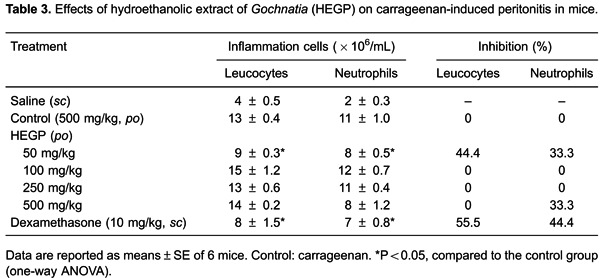

### Effects of HEGP on the secretion of inflammatory mediators

**Figure 3:**
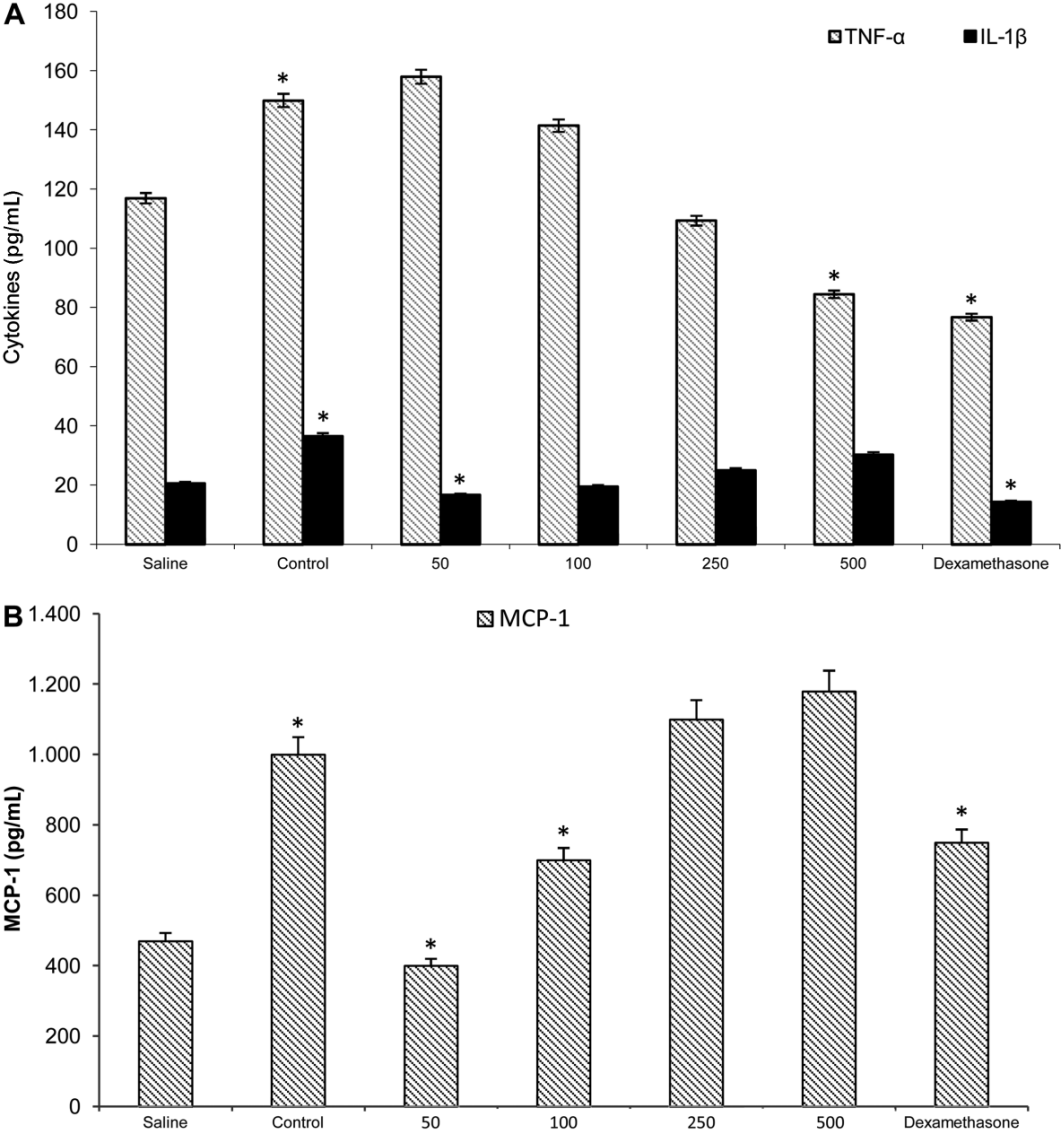
Effects of oral pretreatment with the hydroethanolic extract of *Gochnatia pulchra* (HEGP; 50, 100, 250, and 500 mg/kg) or dexamethasone (10 mg/kg) on carrageenan-induced TNF-α and IL-1β (*A*) and MCP-1 (*B*) production. Data are reported as means±SE of 6 animals. In the control, carrageenan stimulation increased neutrophil secretion in the supernatant after 4 h. *P<0.05, compared to the saline control (one-way ANOVA).

Assessment of the TNF-α, IL-1β, and MCP-1 levels in cell supernatants was performed to investigate whether HEGP can modify the secretion of inflammatory mediators by carrageenan-stimulated neutrophils. As expected, carrageenan stimulation increased the neutrophil secretion of these mediators into the supernatants after 4 h (Figure 3A and B). Treatment with HEGP influenced the secretion of the chemical mediators under basal or carrageenan-stimulated conditions.

## Discussion

Among several traditional claims, literature studies have highlighted that several *Gochnatia* species are useful for the treatment of infections, inflammation, and pain. However, this plant has not yet undergone systematic pharmacological screening. We believe that investigation of these medicinal properties might scientifically authenticate the traditional claims. A previous study reported that the *Gochnatia* genus exhibits selective antibacterial and anti-inflammatory activities, but few papers exist on the phytochemical nature of this genus. To date, scientists have only investigated 14 *Gochnatia* species and isolated 144 compounds from them, comprising 53 sesquiterpene lactones, 10 dimeric lactones, 13 sesquiterpenes, 13 diterpenes, 21 triterpenoids, 20 flavonoids, 7 coumarins, 3 phenolic compounds, 1 acetylenic compound, 1 terpenoid, 1 lignin, and 1 amino acid (4).

During the susceptibility tests for antimicrobial activity, HEGP and the four analyzed fractions showed low activity against *E. faecalis*, *E. coli*, *S*. Choleraesuis, and *P. aeruginosa*. DF was a little more active against *S. aureus* (200 μg/mL). The isolated compound genkwanin displayed the best activity against *S. pyogenes* (100 µg/mL) and *E. faecalis* (25 µg/mL). None of the compounds exhibited activity against the assessed Gram-negative bacteria under the concentrations and conditions tested. The tested compounds displayed similar actions against individual microorganisms, possibly because they had similar structures and therefore similar structure-activity relationships.

HEGP inhibited carrageenan-induced edema formation and protein extravasation, suggesting that its extracts could exhibit anti-inflammatory effects. Indeed, HEGP (50 mg/kg) inhibited leukocyte migration, an important contribution to its anti-inflammatory activity. Phytochemical studies showed that HEGP contained the following major components: 3,5-O-dicaffeoylquinic acid; flavonoids genkwanin, scutellarin, and apigenin; and triterpenoids β-amyrin, taraxasterol acetate, lupeol, and lupeol acetate.

Researchers have tested many plant-derived compounds to clarify whether their anti-inflammatory activity stemmed from an ability to block leukotriene synthesis in rat, mouse, and human cells. Some plant-derived chemical constituents like flavonoids, coumarins, quinones, pentacyclic triterpenes, sesquiterpenes, alkaloids, and polyacetylates can inhibit 5-lipoxygenase (20,21).

Carrageenan injection elicits an exudate in the pleural cavity (22,23) and leukocyte migration (22). This method of inducing inflammation is quite interesting, because it allows assessment of the inflammatory infiltrate and confirms rat paw edema results. Non-steroidal anti-inflammatory drugs, such as indomethacin and dexamethasone, inhibit exudate accumulation and leukocyte mobilization 3-6 h after carrageenan application (24). Inflammation is a protective process that is essential for preserving the integrity of the organism in the event of chemical, physical, and infectious damage. In our experiments, intrapleural carrageenan injection elicited an acute inflammatory reaction, characterized by marked accumulation of a volume of pleural exudate, plasma exudation, and intense polymorphonuclear cell migration into the pleural cavity.

By reducing the volume of the exudate and leukocyte migration, HEGP corroborated the results of the paw edema assay (Figure 2). The antiedematogenic activity of HEGP appeared to be dose-dependent. The components of the extract possibly inhibited prostaglandin biosynthesis, similar to the case for indomethacin and dexamethasone (25).

The immune system produces TNF-α, a pleotropic inflammatory cytokine that suppresses tumor cell proliferation. As studies have established that TNF-α is a key mediator of inflammation (26,27), it is an important parameter to consider when determining the anti-inflammatory activity of plant extracts. Furthermore, IL-1, an important cytokine produced by blood monocytes, mediates the panoply of host reactions collectively known as the acute phase response, and is also known as an endogenous pyrogen, mononuclear cell factor, and lymphocyte-activating factor. TNF-α and IL-1β are potent proinflammatory cytokines that can induce multiple signaling cascades. These cascades act during host defense and paradoxically contribute to inflammatory tissue injury (28). Both IL-1α and IL-1β can trigger fever by stimulating the vascular endothelium of the hypothalamus to synthesize prostaglandin E2, and can also prompt T-cell proliferation (29). The results of the present study revealed that HEGP affected IL-1β and TNF-α levels more significantly than it affected MCP-1 levels. This extract significantly suppressed IL-1β and TNF-α secretion, and its effects resembled the effects of the standard drug dexamethasone. Regarding evaluation of the IL-1β and MCP-1 levels, HEGP (50 mg/kg) elicited less pronounced effects than the control, indicating that HEGP at this concentration exerted an anti-inflammatory action.

Carrageenan is a strong chemical that causes a reproducible inflammatory reaction and the release of inflammatory and proinflammatory mediators (prostaglandins, leukotrienes, histamine, bradykinin, and TNF-α) (30). The components of inflammation include leukocyte infiltration and fluid accumulation, which accompany the cardinal signs of inflammation such as heat, swelling, redness, and pain. Carrageenan remains the standard irritant for examining acute inflammation and the effects of anti-inflammatory drugs (13). Carrageenan-induced inflammation develops immediately after subcutaneous injections and results from the combined actions of prostaglandins, bradykinin, histamine, tachykinins, and reactive oxygen species. Neutrophils readily migrate to the sites of inflammation (31). In the present study, HEGP significantly decreased paw edema and leukocyte infiltration. These effects were similar to those exhibited by the rats treated with indomethacin and dexamethasone. In rats, the carrageenan-induced inflammatory process involves the release of histamine, serotonin, bradykinin, and prostaglandins (32). Many mediators, including lipids, proteinases, biogenic amines, and peptides, participate in this process (33).

Despite numerous studies on the mechanisms underlying the anti-inflammatory properties of HEGP, the role of HEGP in neutrophil trafficking from blood into inflamed tissues still requires further investigation. Here we showed that HEGP affected neutrophil migration, which directly modified the neutrophil adhesive and locomotory functions, especially those related to their rolling behavior, adhesion, and oriented locomotion.

IL-1β plasma levels appear to reflect changes in inflammation (34). Indeed, IL-1β is an important immune mediator that coordinates the activity of different immune cells with vital roles in the acute phase response (35). Administration of the *G. pulchra* extract abated the carrageenan-induced elevations in TNF-α, IL-1β, and MCP-1 levels. HEGP at doses of 50 and 100 mg/kg significantly decreased MCP-1 levels, and higher HEGP doses may stimulate expression of this mediator. HEGP exerted practically the same effect as the reference drug dexamethasone, meaning that HEGP effectively diminished inflammation.

The course of acute inflammation is biphasic. The first phase starts with the release of histamine, serotonin, and kinins during the first few hours after phlogistic agent injection (36). The second phase involves the release of prostaglandin-like substances at 2-3 h after phlogistic agent injection and is sensitive to clinically useful steroidal and non-steroidal anti-inflammatory agents (14). Prostaglandins are the main culprits in acute inflammation. *G. pulchra* might contain an anti-inflammatory agent that blocks the prostaglandin inflammatory pathway.

The anti-inflammatory activities reported for the identified compounds (37,38) may explain the results obtained for the EF fraction. However, we cannot rule out that the activity of this fraction could be caused by a very minor compound that still requires identification. Therefore, it is important to determine the specific compound(s) accounting for the antimicrobial and anti-inflammatory activities of *G. pulchra* and to establish the mechanism of action of its extract before reaching a definitive conclusion.

The present results indicated that HEGP possesses potential anti-inflammatory effects. DF and the flavonoids genkwanin, scutellarin, and apigenin exert antibacterial effects, suggesting that they have potential for application to therapeutic purposes. These results may account for the use of *G. pulchra* in folk medicine. Further studies are necessary to ensure the safe use of this plant extract and its isolated compounds.
